# Validation of a pharmacological imaging challenge using ^11^C-buprenorphine and ^18^F-2-fluoro-2-deoxy-D-glucose positron emission tomography to study the effects of buprenorphine to the rat brain

**DOI:** 10.3389/fnins.2023.1181786

**Published:** 2023-05-10

**Authors:** Amélie Soyer, Sarah Leterrier, Louise Breuil, Maud Goislard, Claire Leroy, Wadad Saba, Karine Thibault, Gregory Dal Bo, Michel Bottlaender, Fabien Caillé, Sébastien Goutal, Nicolas Tournier

**Affiliations:** ^1^Laboratoire d’Imagerie Biomédicale Multimodale (BioMaps), CEA, CNRS, Inserm, Service Hospitalier Frédéric Joliot, Université Paris-Saclay, Orsay, France; ^2^Department of Toxicology and Chemical Risks, Armed Forces Biomedical Research Institute, Bretigny sur Orge, France

**Keywords:** addiction, neuroimaging, opioid, PET imaging, pharmacodynamics, pharmacokinetics, receptor occupancy, neuroreceptor imaging

## Abstract

**Aim:**

Buprenorphine mainly acts as an agonist of mu-opioid receptors (mu-OR). High dose buprenorphine does not cause respiratory depression and can be safely administered to elicit typical opioid effects and explore pharmacodynamics. Acute buprenorphine, associated with functional and quantitative neuroimaging, may therefore provide a fully translational pharmacological challenge to explore the variability of response to opioids *in vivo*. We hypothesized that the CNS effects of acute buprenorphine could be monitored through changes in regional brain glucose metabolism, assessed using ^18^F-FDG microPET in rats.

**Materials and methods:**

First, level of receptor occupancy associated with a single dose of buprenorphine (0.1 mg/kg, s.c) was investigated through blocking experiments using ^11^C-buprenorphine PET imaging. Behavioral study using the elevated plus-maze test (EPM) was performed to assess the impact of the selected dose on anxiety and also locomotor activity. Then, brain PET imaging using ^18^F-FDG was performed 30 min after injection of unlabeled buprenorphine (0.1 mg/kg, s.c) vs. saline. Two different ^18^F-FDG PET acquisition paradigms were compared: (i) ^18^F-FDG injected i.v. under anesthesia and (ii) ^18^F-FDG injected i.p. in awake animals to limit the impact of anesthesia.

**Results:**

The selected dose of buprenorphine fully blocked the binding of ^11^C-buprenorphine in brain regions, suggesting complete receptor occupancy. This dose had no significant impact on behavioral tests used, regardless of the anesthetized/awake handling paradigm. In anesthetized rats, injection of unlabeled buprenorphine decreased the brain uptake of ^18^F-FDG in most brain regions except in the cerebellum which could be used as a normalization region. Buprenorphine treatment significantly decreased the normalized brain uptake of ^18^F-FDG in the thalamus, striatum and midbrain (*p* < 0.05), where binding of ^11^C-buprenorphine was the highest. The awake paradigm did not improve sensitivity and impact of buprenorphine on brain glucose metabolism could not be reliably estimated.

**Conclusion:**

Buprenorphine (0.1 mg/kg, s.c) combined with ^18^F-FDG brain PET in isoflurane anesthetized rats provides a simple pharmacological imaging challenge to investigate the CNS effects of full receptor occupancy by this partial mu-OR agonist. Sensitivity of the method was not improved in awake animals. This strategy may be useful to investigate de desensitization of mu-OR associated with opioid tolerance *in vivo*.

## 1. Introduction

Strong mu-opioid receptor (MOR) agonists form the most powerful class of analgesic drugs. Their clinical management is however difficult due to several side effects and to significant risk for respiratory depression and overdose (Trescot et al., 2008). Prolonged use of opioids is associated with tolerance and addictive properties which may lead to misuse and drug abuse as exemplified by the current opioid crisis (Volkow and Collins, 2017; Volkow and Blanco, 2021).

There is a crucial need for *in vivo* methods to explore the molecular determinants of opioid response, tolerance and dependence in animals and humans (Morgan and Christie, 2011). In neuropharmacology, pharmacological challenges are essential to explore the pharmacokinetics (PK) and pharmacodynamics (PD) of opioids in animals and humans. However, management of pharmacological challenges using strong opioid may be poorly tolerated and appear risky given the high variability in terms of side effects experienced, which is difficult to predict, especially in healthy volunteers (Drewes et al., 2013).

Among strong opioid drugs, buprenorphine (BUP) benefits from a unique safety profile (Wakeman and Barnett, 2018). *In vitro*, buprenorphine is a high affinity agonist of MOR but is also described as antagonist of κ-OR, δ-OR, and agonist of nociceptin/ORL-1 receptors (Lutfy and Cowan, 2004; Cami-Kobeci et al., 2011). *In vivo*, buprenorphine mainly acts as a high-affinity and high-potency partial agonist (i.e., intermediate intrinsic efficacy) of MOR, although affinity for other opioid receptor subtypes may account for its neuropharmacological profile (Infantino et al., 2021). Low-dose buprenorphine offers potent analgesia for the treatment of moderate to severe pain in patients (Lutfy and Cowan, 2004). High dose buprenorphine, which is associated with almost full MOR occupancy in humans (Greenwald et al., 2003), does not cause respiratory depression and can be safely administered for maintenance therapy in patients with opioid use disorder (Helm et al., 2008; Wakeman and Barnett, 2018). A “ceiling” analgesic dose–response has been described in animals, suggesting limited efficacy of buprenorphine at stimulating MOR, which refers to a partial agonist profile. However, in humans, buprenorphine displays ceiling in respiratory effect but none in analgesic effect, thus behaving as a full agonist (Dahan et al., 2006). Because of buprenorphine’s high MOR affinity, its acute effects may drastically depend on the baseline pharmacologic conditions and individual differences in MOR availability and signaling (Greenwald et al., 2022). Single dose buprenorphine can therefore be considered a safe pharmacological challenge to safely explore individual differences in the effects of high level of MOR occupancy in animals and humans.

Conventional PK/PD studies offer limited insight into the impact of opioids at the CNS level, which is essential to understand the brain mechanisms involved in their neuropharmacology (Ing Lorenzini et al., 2012). In this framework, functional neuroimaging techniques such as positron emission tomography (PET) or functional magnetic resonance imaging (fMRI) offer minimally-invasive methods to investigate the impact of opioids on brain function in animals and humans (Cumming et al., 2019; Moningka et al., 2019). PET imaging using the MOR-agonist ^11^C-carfentanyl has been extensively used to estimate the target engagement associated with clinical doses of MOR ligands including buprenorphine (Cumming et al., 2019). Recent preclinical studies using carbon-11 radiolabeled buprenorphine (^11^C-buprenorphine) in rats and non-human primates have been reported as a method to simultaneously explore the neuropharmacokinetics of buprenorphine and its specific neuroreceptor binding, mainly at MORs (Auvity et al., 2021). Pharmacological functional MRI (phMRI) has been validated in animals and humans to describe the effects of buprenorphine on brain function (Upadhyay et al., 2011; Becerra et al., 2013; Seah et al., 2014). However, this preclinical work raised important questions regarding the impact of anesthesia which abolished the significance of phMRI measurements in response to buprenorphine (Seah et al., 2014).

In the present study, we hypothesized that the CNS effects of full receptor occupancy by buprenorphine could be safely estimated through changes in brain glucose metabolism in rats, assessed using ^18^F-2-fluoro-2-deoxy-D-glucose (^18^F-FDG) PET imaging. First, a dose of buprenorphine with minimal effects on animal behavior and full receptor occupancy was validated. Then, ^18^F-FDG PET imaging was performed in anesthetized animals or using an alternative acquisition protocol which aimed at limiting the impact of anesthesia on the outcome of brain PET data.

## 2. Materials and methods

### 2.1. Chemicals, radiochemicals

Buprenorphine hydrochloride for injection (0.1 mg/mL) was obtained from Coveto (France). ^18^F-2-fluoro-2-deoxy-D-glucose (^18^F-FDG) for injection was obtained from Cyclopharma (France). Isoflurane was obtained from Abbvie (Rungis, France). ^11^C-Buprenorphine was synthesized in-house from 3-*O*-triyl-6-*O*-desmethylbuprenorphine (ABX, Germany) in two steps using a C-11 Pro-2 module (iPhase Technologie, Australia). Ready-to-inject ^11^C-buprenorphine (1.8–3.5 GBq, decay-corrected radiochemical yield:15%) was obtained with a radiochemical purity above 98% and a mean molar activity of 93 ± 10 GBq/μmol EOB (End of bombarding).

### 2.2. Animals

Thirty-two male Sprague–Dawley rats were used for this study. Eighteen rats were used for PET imaging experiments. Different 14 rats were used for behavioral experiments. Rats were housed under standard experimental conditions: room temperature (20 ± 2°C); light/dark cycle (14 h light/10 h dark); water and food *ad libitum* and kept in social groups of two rats per cage. An acclimation period of 7 days was respected before any experiment. All procedures were in accordance with European directives on the protection and use of laboratory animals (Council Directive 2010/63/UE, French decree 2013–118). The experimental protocol was evaluated and validated by a local ethic committee for animal use and approved by the French government (n° APAFIS#34694–2,022,011,718,132,961 v1). Anaesthesia was induced and maintained using isoflurane 2–2.5% in O_2_.

### 2.3. MicroPET acquisition

Brain microPET acquisitions were performed in anesthetized rats using an Inveon, microPET (Siemens Healthcare, Knoxville, TN, United States, spatial resolution 1.6 mm).

### 2.4. ^11^C-Buprenorphine PET study

First, level of receptor occupancy associated with the selected dose of buprenorphine (0.1 mg/kg, s.c) was investigated using ^11^C-buprenorphine PET imaging in anesthetized rats. Rats were s.c injected with unlabeled buprenorphine (0.1 mg/kg (*n* = 3)), 10 mg/kg (*n* = 2) or vehicle (Saline vehicle, *n* = 3). After 30 min, ^11^C-buprenorphine was i.v injected followed by dynamic PET acquisition.

### 2.5. ^18^F-FDG PET study

#### 2.5.1. Anesthetized rat paradigm

First, awake rats received either s.c injection of buprenorphine (0.1 mg/kg s.c., *n* = 5) or vehicle (*n* = 5). After 25 min, anaesthesia was induced using isoflurane 2–2.5% in O_2_ and a catheter was inserted in the caudal vein. At 30 min, ^18^F-FDG was i.v injected (37.62 ± 2.3 MBq), followed by 30 min static PET acquisition starting after 20 min of ^18^F-FDG uptake (20–50 min).

#### 2.5.2. Awake rat paradigm

A customized PET protocol was used to limit the impact of anaesthesia on the estimation of brain function using ^18^F-FDG PET (Schiffer et al., 2007). This protocol takes advantage of the irreversible uptake of ^18^F-FDG by the brain. Thirty min after s.c injection of buprenorphine (0.1 mg/kg s.c. *n* = 4) or vehicle (*n* = 4) in awake animals, ^18^F-FDG (1 ml; mean dose = 33.6 ± 4.1 MBq) was injected intraperitoneally (i.p), in awake rats. Then, rats were then transferred in a cage to allow for the brain uptake of ^18^F-FDG in awake and freely moving animals. It takes 30 min for i.p administered ^18^F-FDG to reach the plateau brain uptake of i.v administered ^18^F-FDG (Schiffer et al., 2007). Therefore, after 35 min uptake, anaesthesia was induced before transferring rat to the microPET scanner for a 30 min static PET acquisition (40–70 min).

#### 2.5.3. PET data reconstruction and analysis

PET images were reconstructed by the 2D OSEM/FORE algorithm and corrected for attenuation, random coincidences and scatter. The voxel size was 0.2 × 0.2 × 0.2 mm^3^. Brain PET images were corrected for radioactive decay, injected dose and body weight and was expressed as standardized uptake values (SUV), with SUV = tissue activity (kBq/cc)/[injected dose (kBq)/body weight (g)]. SUV-normalized PET images were spatially co-registered to a standard rat ^18^F-FDG PET template using Pmod software (version 4.2, PMOD Technologies Ltd., Zurich, Switzerland). Quantitative SUV values were determined through a volume-of-interest (VOI) analysis in selected brain regions which size is relevant to the spatial resolution of the microPET scanner (~1.6 mm) which included thalamus, cerebellum, striatum, hippocampus, hypothalamus, midbrain, septum, ventral tegmental area (VTA), pons and cortex.

For dynamic ^11^C-buprenorphine PET images, SUV-normalized time-activity curves were obtained in selected brain regions. Summed PET images from 40–60 min were generated for regional analysis. Regional SUV values were obtained from static ^18^F-FDG PET images.

For both the ^18^F-FDG and the ^11^C-buprenorphine PET images, regional SUV values were then divided by respective SUV values in the cerebellum to obtain SUVR.

### 2.6. Anxiety and locomotor activity

We hypothesized that animal handling to perform i.p injection of ^18^F-FDG in awake rat may impact animal behavior with consequences for the pharmacological imaging challenge. The impact of animal handling and/or pre-treatment by unlabeled buprenorphine on animal anxiety and activity was investigated using the elevated plus-maze (EPM) test (Etaee et al., 2017). Rats were assigned to 4 groups (*n* = 7 in per group) which recapitulated buprenorphine treatment and the type of ^18^F-FDG injection. Rats used to simulate the awake paradigm did not require anaesthesia and the EPM was performed 40 min after i.p injection of 0.5 mL saline. Animals used to simulate the anesthetized paradigm underwent the EPM test 20 min after the end of anaesthesia during which a venous catheter was inserted and 1 mL saline was injected. After treatment by buprenorphine 0.1 mg/kg or vehicle (saline), each rat was installed at the center of the maze and allowed to explore all arms freely. Behavior was controlled for 5 min over the maze using a dedicated digital camera. The maze was cleaned after each experiment in each animal. The time spent in the open arms, as well as the total distance covered by the rat were determined.

### 2.7. Statistical analysis

A two-way ANOVA with Turkey’s *post hoc* test was performed to compare SUV or SUVR values between groups for each brain regions. Statistical analysis was performed using GraphPad software (version 9.0).

## 3. Results

### 3.1. ^11^C-Buprenorphine PET study

**Figure 1:**
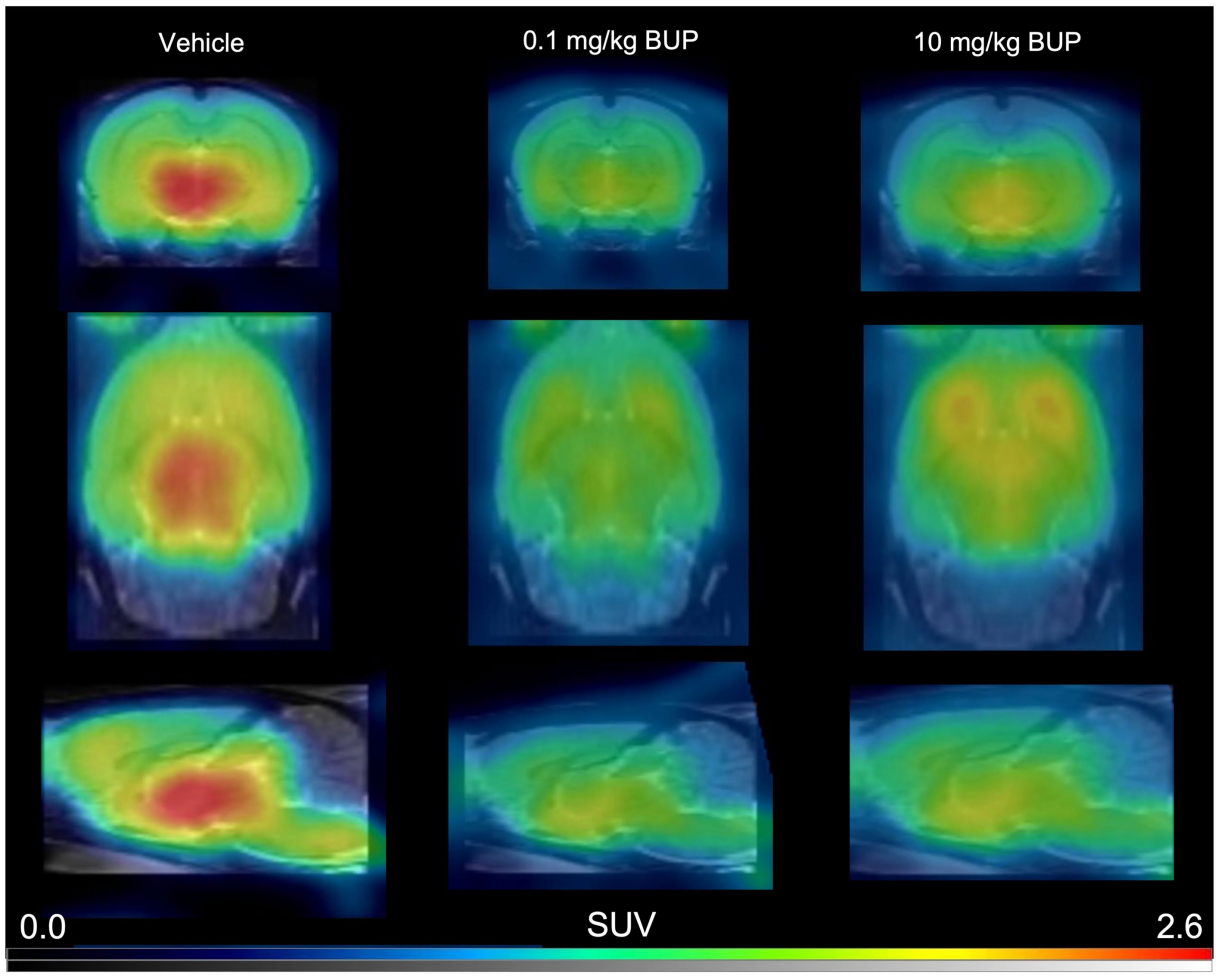
Representative ^11^C-buprenorphine PET images. These are summed PET images (40–60 min) obtained in the rat brain without (vehicle) or after pharmacological blocking using unlabelled buprenorphine (BUP) 0.1 mg/kg or 10 mg/kg injected s.c 30 min before ^11^C-buprenorphine.

**Figure 2:**
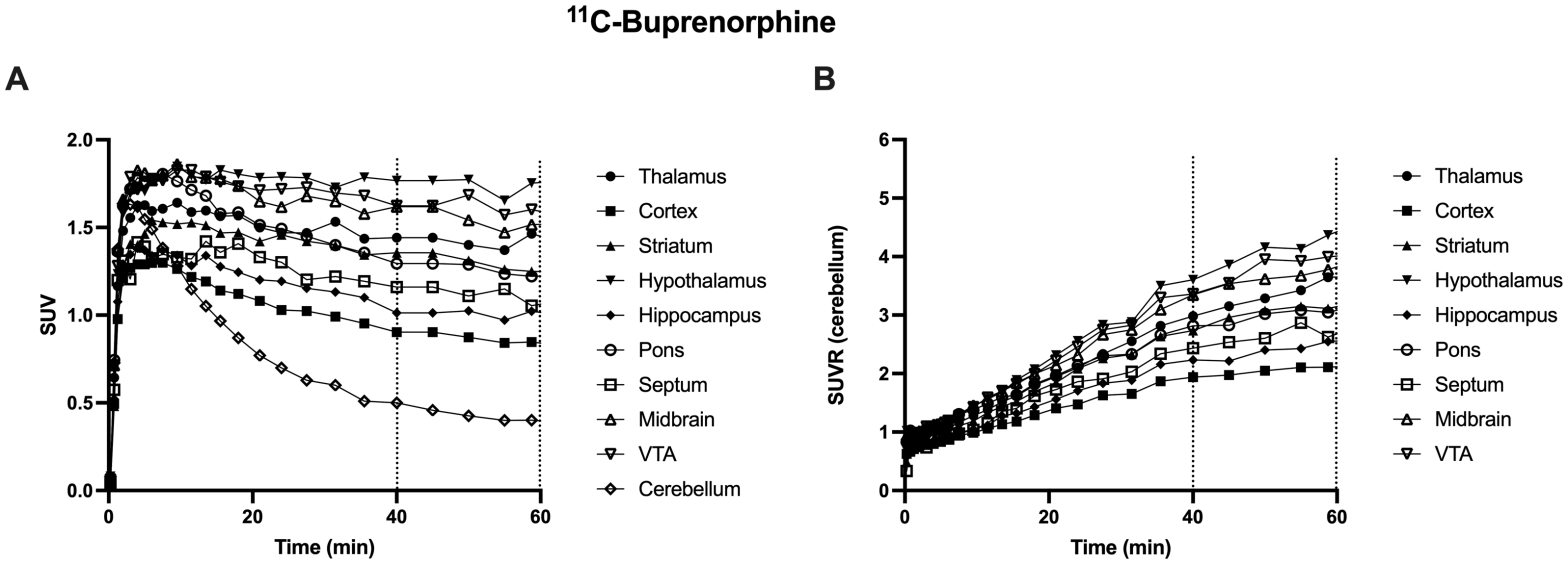
Representative brain kinetics of ^11^C-buprenorphine in selected brain regions in rats. In **A**, data are expressed as standardized uptake value (SUV) versus time (min). Corresponding data, normalized by SUV values in the cerebellum (SUVR) are shown in **B**. Dashed lines show the time frame used for ^11^C-buprenorphine PET data analysis (from 40 to 60 min) VTA: ventral tegmental area.

**Figure 3:**
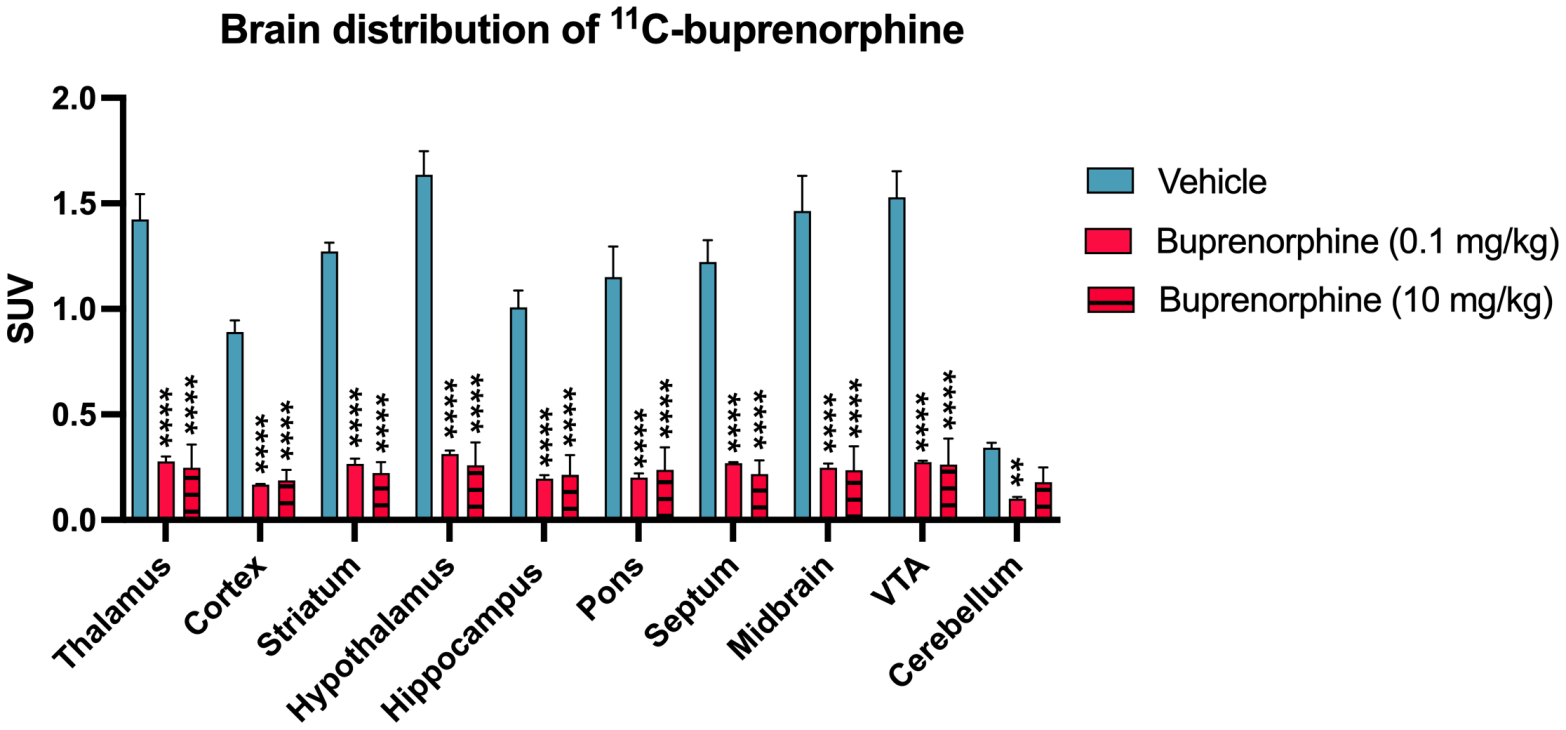
Impact of pharmacological blocking using unlabelled buprenorphine on the regional brain uptake of ^11^C-buprenorphine uptake of ^11^C-buprenorphine. Data are expressed as standardized uptake value (SUV) and reported as mean ± S.D. obtained from summed (40–60 min) PET images obtained in the rat brain without (vehicle) or after pharmacological blocking using unlabelled buprenorphine (BUP) 0.1 mg/kg or 10 mg/kg injected s.c 30 min before ^11^C-buprenorphine. VTA: ventral tegmental area. Statistical significance was set at *p* < 0.05 with ^**^p < 0.01 and ^****^p < 0.0001.

In control rats, s.c injected with saline 30 min before PET, brain distribution of microdose ^11^C-buprenorphine was consistent with previous report (Auvity et al., 2021; Vodovar et al., 2022). SUV-normalized PET images showed higher uptake in subcortical brain regions compared with the cortex (Figure 1). Significant uptake by these regions contrasted with the very low uptake of ^11^C-buprenorphine by the cerebellum (Figures 2, 3). Time-activity curves obtained in the different brain regions showed a relatively slow uptake of ^11^C-buprenorphine, which peaked at ~7 min, followed slow washout of brain radioactivity (Figure 2A). The lowest uptake was found in the cerebellum which is assumed to be devoid of MOR in rats (Tempel and Zukin, 1987) (Figures 2, 3). Pseudo-equilibrium of concentration of ^11^C-buprenorphine in brain regions relative to the cerebellum was almost reached at ~40 min (Figure 2B). Summed PET images (40–60 min) were therefore used for further analyses as a compromise between pseudo-equilibrium and the short radioactive half-life of carbon-11 (20.4 min), to allow for quantitative determination of the PET signal in brain regions, including the cerebellum. Highest SUV values were found in the hypothalamus, midbrain, VTA and thalamus.

Blocking experiment by unlabeled buprenorphine (0.1 mg/kg) visually decreased the brain PET signal (Figure 1). Blocking significantly decreased the binding of ^11^C-buprenorphine in all selected brain regions including, in a lower extent, in the cerebellum. Higher dose of unlabeled buprenorphine (10 mg/kg) did not further block the binding of ^11^C-buprenorphine in any brain region, thus suggesting full receptor occupancy by buprenorphine 0.1 mg/kg (Figure 3).

### 3.2. ^18^F-FDG PET study

**Figure 4:**
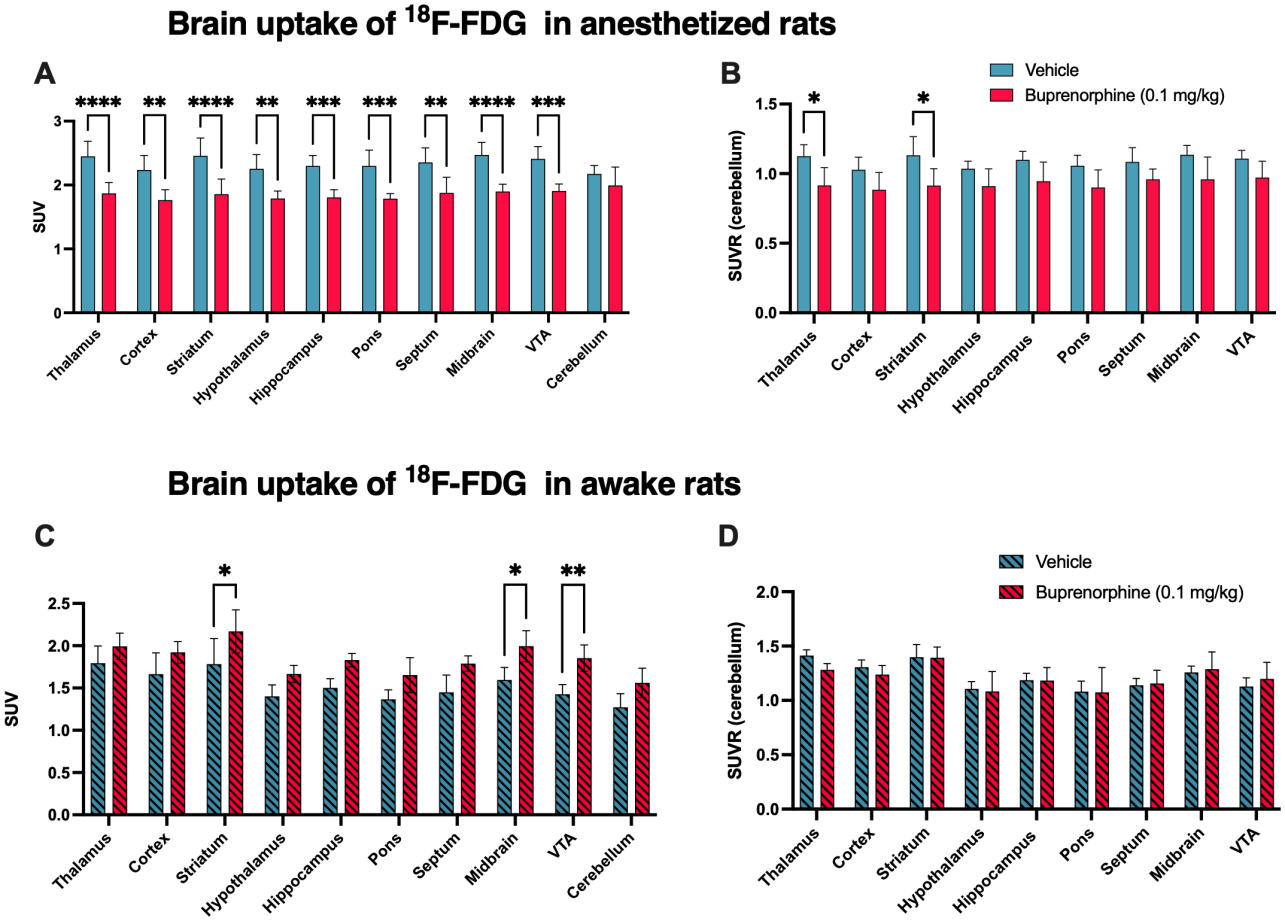
Impact of the buprenorphine challenge on brain function estimated using ^18^F-FDG PET imaging in rats. The uptake of ^18^F-FDG Data in selected brain regions is expressed as standardized uptake value (SUV) and reported as mean ± S.D. (*n* = 4–5 per group). Brain ^18^F-FDG PET data were obtained without (vehicle) or after pharmacological challenge using unlabelled buprenorphine 0.1 mg/kg in anesthetized (in **A**) or in awake animals (in **C**). SUVR corresponds to the regional uptake of ^18^F-FDG in brain region divided by the cerebellar uptake obtained in anesthetized (in **B**) or in awake animals (in **D**). VTA: ventral tegmental area. Statistical significance was set at ^*^p < 0.05 with ^**^p < 0.01, ^***^p < 0.001 and ^****^p < 0.0001.

The impact of full receptor occupancy by buprenorphine 0.1 mg/kg on brain glucose metabolism was then investigated using ^18^F-FDG PET (Figure 4). In anesthetized rats, SUV-normalized ^18^F-FDG PET images revealed a lower ^18^F-FDG uptake in buprenorphine-treated animals compared with control (saline) rats. The decrease in ^18^F-FDG uptake was significant in all brain regions (*p* < 0.01 to <0.0001) except in the cerebellum (*p* > 0.05, Figure 4A). The highest level of significance for the impact of buprenorphine on ^18^F-FDG uptake was observed in the thalamus, striatum and midbrain (Figure 4A). Cerebellum was then used as a normalization region to generate regional SUVRs which confirmed the trend in reduced relative ^18^F-FDG uptake which was significant in the thalamus and striatum (*p* < 0.05) (Figure 4B).

In awake animals, the SUV-normalized uptake of ^18^F-FDG tended to be higher in BUP treated rats compared with vehicle-treated rats, with a similar impact across brain regions, including the cerebellum. Increase in ^18^F-FDG SUV values was significant only in the striatum, midbrain and VTA (p < 0.05, Figure 4C). However, in the awake situation, SUVR values were strikingly similar in buprenorphine-treated and control rats with no significant difference (Figure 4D).

### 3.3. Behavioral test

**Figure 5:**
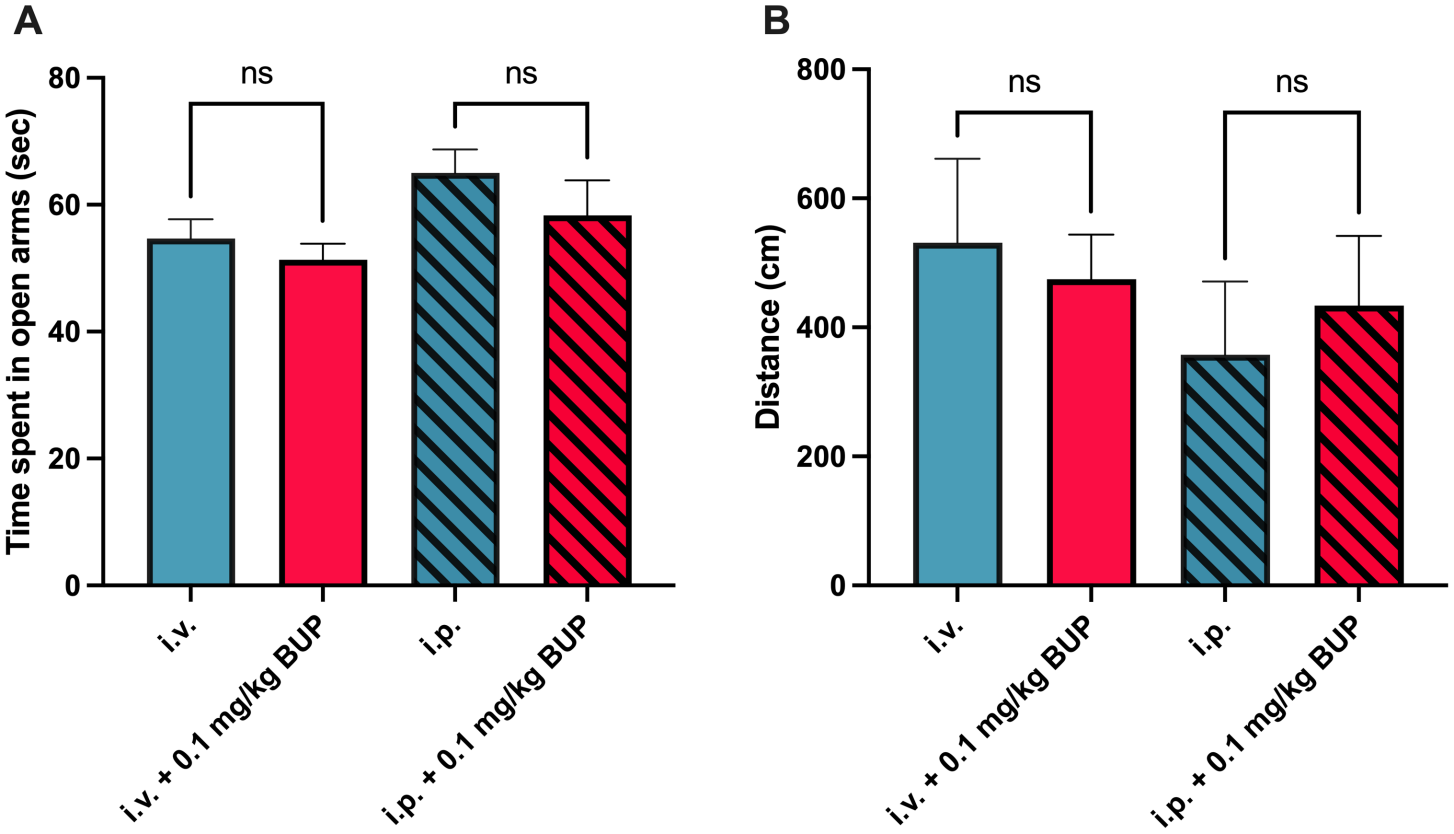
Behavioral analysis for elevated plus maze. Corresponding time spent in open arms (in sec, *n* = 7 per group) in awake paradigm (full bars) and after the anesthetized paradigm (hatched bars) associated with animal handling during the anesthetized paradigm (i.v injection) or awake paradigm, i.p injection) is shown in **A**. This was measured without or with administration of buprenorphine (0.1 mg/kg, BUP). Corresponding locomotor activity in both the open and walled arms was determined (in cm) is shown in **B**. n.s: non-significant.

The EPM test showed that treatment by buprenorphine 0.1 mg/kg did not significantly impact animal behavior in terms of anxiety or locomotor activity (*p* > 0.05, Figure 5). This was similarly observed in both the awake or the anaesthetized paradigm.

## 4. Discussion

Innovative pharmacological challenges associated with appropriate pharmacological neuroimaging techniques are needed to explore the complexity of the neuropharmacology of opioids and combat the opioid pandemic (Volkow and Collins, 2017; Moningka et al., 2019). The aim of this study was to validate a reproducible pharmacological challenge, safely enabling full receptor occupancy of MOR, which CNS effects can be quantitatively monitored using brain ^18^F-FDG PET in rats.

First, we used brain ^11^C-buprenorphine PET imaging in rats to show that full saturation of neuroreceptors was achieved with the challenge dose of buprenorphine (0.1 mg/kg, s.c). Target engagement is better correlated with plasma concentration of buprenorphine rather than injected dose (Auvity et al., 2021). Pharmacological challenge associated with full receptor occupancy is therefore preferable to limit the impact of pharmacokinetic variability, which may lead to different level of receptor occupancy, on PD measurements. The results obtained after injection with a 100-fold higher dose of buprenorphine (10 mg/kg) did not further block the binding of ^11^C-buprenorphine to any brain region confirming that full saturation was achieved with buprenorphine 0.1 mg/kg. Receptor occupancy was estimated using ^11^C-buprenorphine PET imaging rather than a MOR-specific radioligand such as ^11^C-carfentanyl (Eriksson and Antoni, 2015). This enabled investigation of the binding of buprenorphine to any CNS target, which may not restrict to MOR (Lutfy and Cowan, 2004). However, very low binding of ^11^C-buprenorphine in the cerebellum compared with other brain regions is consistent with the low expression of MOR in the rat cerebellum (Tempel and Zukin, 1987), and was similarly observed in nonhuman primates (Galynker et al., 1996; Auvity et al., 2021). This cerebellar binding may either reflect low but significant expression or MOR in the cerebellum, or significant binding of ^11^C-buprenorphine to kappa-opioid receptor (KOR) which are widely expressed in the rat cerebellum (Tempel and Zukin, 1987; Peckys and Landwehrmeyer, 1999). However, the very low involvement of ^11^C-buprenorphine binding to the cerebellum compared with other brain regions supports its predominant binding to MOR, as previously demonstrated using blocking by either MOR-or KOR-specific ligands in rats (Auvity et al., 2021).

Dynamic PET data revealed that maximum brain uptake of ^11^C-buprenorphine was achieved ~10 min after i.v injection (Figure 2). The slow washout of radioactivity from the brain is consistent with the slow dissociation rate of buprenorphine to MOR *in vitro* (Bidlack et al., 2018). These PET data moreover show that buprenorphine binding to neuroreceptor is relatively stable over a prolonged time-frame which ensures stability of the MOR occupancy during pharmacodynamic or pharmacological imaging assessment. Brain ^18^F-FDG PET was therefore injected 30 min after the buprenorphine challenge to capture a time-frame during which MOR are fully occupied.

Buprenorphine-induced hyperactivity has been reported in certain rat strains (Burke et al., 2019). The EPM test was therefore conducted in our Sprague–Dawley rats and showed that the selected dose of buprenorphine did not impact animal behavior in terms of anxiety or locomotor activity. This means that change in brain function, as assessed from the outcome of brain ^18^F-FDG experiments, is not likely to reflect dramatic change in animal behavior or peripheral movement during the buprenorphine challenge in conscious animals. The limited impact of buprenorphine on animal behavior prompted us to test of a paradigm aiming at limiting the impact of anaesthesia on the outcome of brain ^18^F-FDG PET data.

In the anesthetized rat paradigm, unlabelled buprenorphine was injected in awake animal 30 min before ^18^F-FDG PET acquisition, which was performed under isoflurane anaesthesia. The buprenorphine challenge significantly decreased ^18^F-FDG uptake (SUV) in all tested brain region except the cerebellum. This suggests that conventional brain ^18^F-FDG PET, performed under isoflurane anaesthesia, can be used as a translational biomarker of the response to the buprenorphine challenge in the brain. This provides a simple pharmacological imaging paradigm to investigate the CNS effects of full receptor occupancy by this MOR agonist. These preclinical data are consistent with previously reported clinical PET data obtained in polydrug abusers. A single dose of buprenorphine (1 mg, intramuscularly) significantly decreased the brain glucose consumption by 21%, as assessed using brain ^18^F-FDG PET with kinetic modeling, which takes both the arterial input function and blood glucose level into account (Walsh et al., 1994).

Efforts were then made to improve the significance and/or the sensitivity of ^18^F-FDG PET to non-invasively investigate the CNS effects of buprenorphine. First, using the anesthetized rat paradigm, normalization of the regional PET data by the uptake by the cerebellum led to a global decrease in SUVR values in the brain. This strongly suggests that the decrease in ^18^F-FDG uptake by the brain induced by buprenorphine is not due to any change in the peripheral metabolism of glucose or ^18^F-FDG. However, regional difference in SUVR, as tested using post-hoc analysis, was significant only in the thalamus and striatum (*p* < 0.05). This may be explained by the relatively low number of rats in each group (*n* = 5). Moreover, our study used different individuals to compare ^18^F-FDG uptake without and after pre-treatment by buprenorphine, which did not take full advantage of PET imaging as a minimally invasive procedure (Lancelot and Zimmer, 2010; Zimmer, 2023). For future studies, comparison of the regional ^18^F-FDG uptake in the same individuals, before and after administration of buprenorphine, would enable paired comparison with higher statistical power to better capture and compare the effects of buprenorphine across brain regions.

It is often assumed that general anaesthesia may impede the CNS effects of drugs, thus supporting the use of neuroimaging technique in awake and conscious animals (Chang et al., 2016; Cavaliere et al., 2018). This particularly holds for preclinical phMRI studies for which neuroimaging teams have to develop a sophisticated expertise to prevent motion artifacts and reduce restraint-induced stress during acquisition (Ferrari et al., 2012). We hypothesized that the awake rat paradigm, which proved to be useful for pharmacological ^18^F-FDG PET studies in rodents (Levigoureux et al., 2019; Hugon et al., 2022), may offer enhanced sensitivity to detect the CNS effects of buprenorphine. Baseline SUV values in the brain were lower in the awake compared with the anesthetized rat paradigm. In anesthetized rats, limited impact of the injection route (i.v *vs* i.p injection) on late brain SUVs has been reported (Schiffer et al., 2007). Moreover, isoflurane anaesthesia used in the uptake phase of anesthetized rat paradigm, but not in the awake rat paradigm, was expected to decrease ^18^F-FDG uptake by the brain (Spangler-Bickell et al., 2016). Unexpectedly, in awake rats, a global increase in SUV was observed which was significant at the regional level in the striatum, midbrain (*p* < 0.05) and VTA (*p* < 0.01). However, normalization of regional SUVs by the cerebellar uptake strikingly abolished the global and regional effect of buprenorphine. This suggests that the increase in SUV may rather be due to an alteration in the peripheral metabolism of ^18^F-FDG. Our behavioral data suggest that this may not be linked with change in locomotor activity or anxiety in awake animal. It may be hypothesized that in the awake rat paradigm, buprenorphine may decrease the uptake of ^18^F-FDG by peripheral tissues, thus enhancing its brain availability. Altogether, these results do not support the use of the awake rat paradigm for the pharmacological imaging a buprenorphine in rats, which can be performed using a conventional procedure under isoflurane anaesthesia. The awake paradigm has been successfully used for pharmacological neuroimaging (Levigoureux et al., 2019; Hugon et al., 2022). This means that selection of either the awake or anesthetized paradigm requires preliminary validation and may depend on the investigated drug.

There are several limitations in this study. First, PET data have only been interpreted in the light of SUV and SUVR values, calculated from brain PET data only. Full kinetic modeling, which requires arterial blood sampling, is difficult to perform in rodents and has not been performed in neither the ^11^C-buprenorphine nor the ^18^F-FDG PET study. Buprenorphine is mainly metabolized into norbuprenorphine in rats (Yassen et al., 2012). However, it was shown that norbuprenorphine (Alhaddad et al., 2012), as well as ^11^C-norbuprenorphine (Auvity et al., 2020), negligibly cross the blood–brain barrier compared with parent buprenorphine/^11^C-buprenorphine. It can therefore be assumed that the brain PET signal associated with ^11^C-buprenorphine injection predominantly corresponds to parent, unmetabolized ^11^C-buprenorphine. Similarly, full kinetic modeling would have been useful to quantitatively address the effects of buprenorphine on the brain glucose metabolism using ^18^F-FDG PET. However, in anesthetized rat, the limited impact of buprenorphine on ^18^F-FDG uptake in the cerebellum enabled normalization of the brain PET signal. The cerebellum cannot be properly considered a reference region for ^18^F-FDG because it shows substantial brain uptake. However, normalization of PET data by the cerebellum uptake (SUVR) limits the possible impact of buprenorphine on blood glucose and peripheral kinetics of ^18^F-FDG for correct interpretation of brain PET data.

Our pharmacological imaging data for buprenorphine, obtained using ^18^F-FDG PET imaging, can be directly compared with buprenorphine challenge performed using phMRI and the same dose of buprenorphine in rodents (0.1 mg/kg i.v.). In conscious rats (but not in anesthetized rats), buprenorphine yielded phMRI activation, as assessed from change in the BOLD (blood-oxygen-level dependent) signal in cortex (i.e., cingulate, somatosensory cortex, and insula), subcortical structures (i.e., caudate-putamen, hippocampus, and thalamus) and cerebellum (Becerra et al., 2013). This activation profile corresponds with the brain distribution of ^11^C-buprenorphine except in the cerebellum. Moreover, phMRI deactivation was observed in the cingulate, mammillary nuclei, amygdala, dentate gyrus and brainstem. This complex phMRI response profile contrasts with our ^18^F-FDG PET data showing a decrease in brain glucose metabolism in MOR-rich regions in anesthetized rodent and humans (Walsh et al., 1994). Similar phMRI response profile, with predominant activation pattern, have been reported in awake nonhuman primates (Seah et al., 2014) and humans (Upadhyay et al., 2011), as assessed from change in CBV (cerebral brain volume). These discrepancies suggest that phMRI and ^18^F-FDG PET do not capture the same neuropharmacological parameters to describe the CNS effects of buprenorphine. It may be hypothesized that phMRI captures the direct activation of MORs whereas ^18^F-FDG PET captures the downstream MOR-related effects of buprenorphine which involves modulation of GABA-and glutamate-mediated processes, and results in a global decrease in neuronal activity (Reeves et al., 2022). It can therefore be assumed that ^18^F-FDG PET may reflect the outcome of fMRI data showing the global decrease in functional connectivity observed during a buprenorphine challenge in humans (Upadhyay et al., 2011). Finally, compared with fMRI, PET is a quantitative imaging modality, although only semi-quantitative analysis, based on the determination of SUVR in a limited number of rats, was performed in this study to compare the effect of full receptor occupancy by buprenorphine on brain function (Tournier et al., 2021). Further experiments are needed to explore the ability of ^18^F-FDG PET to distinguish different level of response to the buprenorphine challenge validated here.

## 5. Conclusion

In the present study, a pharmacological imaging challenge using buprenorphine 0.1 mg/kg was validated. This dose enabled full receptor occupancy with limited impact on rat locomotor activity or anxiety. CNS effects induced by this buprenorphine challenge can be monitored using ^18^F-FDG PET in anesthetized rats. This multi-tracer approach using minimally-invasive PET imaging could be combined with phMRI to provide a multiparametric insight into the functional determinants of the acute response to opioids. This may help untangling the neurophysiology of opioid tolerance and dependence *in vivo*.

## Data availability statement

The raw data supporting the conclusions of this article will be made available by the authors, without undue reservation.

## Ethics statement

The animal study was reviewed and approved by CETA n° APAFIS#34694-2022011718132961 v1.

## Author contributions

CL, WS, KT, and NT designed the study. AS, SL, and MG acquired the data. AS, LB, CL, KT, and SG analysed the data. FC supervised the radiochemistry. CL, KT, GB, and MB contributed to the interpretation of the results. AS wrote the first draft. All authors contributed to the article and approved the submitted version.

## Funding

This work was funded by Amélie Soyer received a PhD grant from the CEA (commissariat à l’énergie atomique et aux énergies alternatives) and the AID (Agence de l’innovation de défense - Ministère des Armées). This work was performed on a platform member of France Life Imaging network (grant ANR-11-INBS-0006).

## Conflict of interest

The authors declare that the research was conducted in the absence of any commercial or financial relationships that could be construed as a potential conflict of interest.

## Publisher’s note

All claims expressed in this article are solely those of the authors and do not necessarily represent those of their affiliated organizations, or those of the publisher, the editors and the reviewers. Any product that may be evaluated in this article, or claim that may be made by its manufacturer, is not guaranteed or endorsed by the publisher.
